# Extending wave propagation along muscle fibers originates from early local contraction at the end-plate region

**DOI:** 10.1016/j.jphyss.2025.100023

**Published:** 2025-05-09

**Authors:** Tomonori Hayashi, Naoya Nakahara, Shigeru Morimoto, Maki Yamaguchi, Kazuhiro Hirano, Shigeru Takemori

**Affiliations:** Department of Molecular Physiology, The Jikei University School of Medicine, 3-25-8 Nishishinbashi, Minato-ku, Tokyo 105-8461, Japan

**Keywords:** Extending wave, Stress wave, Twitch, Motor unit, Action potential

## Abstract

We investigated the propagation of extending waves along twitching muscle fibers triggered by the internal shortening of early local contraction in the end-plate region. Bullfrog sartorius muscles were minimally stimulated, and the displacement of carbon particles attached to the muscle surface was captured using a high-speed camera. We found an extending wave along the fiber at a velocity of 5.35 ± 0.33 m·s⁻¹, faster than the conduction of action potentials at 3.04 ± 0.31 m·s⁻¹. Local compression of the muscle surface blocked the propagation of the extending wave, indicating its mechanical nature. Muscle stretching increased the extending wave velocity. These findings provide direct evidence that mechanically transmitted extending waves originate from early local contractions in the end-plate region and propagate along muscle fibers ahead of the contraction wave.

## 1 Introduction

The physiological contraction of muscle fibers in vivo originates from the action of innervating motor neurons at the end-plate region, where the propagation of action potentials diverges toward both fiber ends. The propagated action potential locally spreads into the fibers through the transverse tubular (T-tubule) network and triggers Ca^2+^ release from the subsarcolemmal and intermyofibrillar sarcoplasmic reticulum. The released Ca^2+^ diffuses into the myofibrils to activate the contractile interaction between myosin heads protruding from thick filaments and actin on the thin sarcomere filaments. These processes cause a local distribution of contractile activation levels in the fiber, particularly regarding twitch contraction. It is generally accepted that T-tubules contribute to diminishing uneven activation across the fiber because the propagation of electrical potentials through the T-tubules is significantly faster than Ca^2+^ diffusion in the myoplasm [1], [2]. Regarding the longitudinal distribution of the activation levels, the propagation of sarcolemmal action potential along the fiber is faster than the propagation of electrical potentials through the T-tubules [3], [4].

Morimoto and coworkers reported that the conduction of action potentials from the end-plate region to the distal end of muscle fibers in humans was approximately 15–17 ms in the vastus lateralis muscle and that there should be a significant distribution of activation levels from resting to contracting waves along the fiber [5]. Similarly, they reported a faster propagation of mechanical signals along a fiber, which was recorded using a microphone [6], [7], [8], [9]. They postulated that the mechanical signal represents the thinning of the resting part of the fiber owing to lengthening because of internal shortening of early contraction in the end-plate region. They further hypothesize that such mechanical signals originate from a mechanism where local sarcomere contraction generates mechanical tension that stretches longitudinal elastic elements such as connectin/titin and the cytoskeletal system. This tension pulls adjacent Z-lines and sequentially transmits force to neighboring sarcomeres, ultimately propagating along the entire fiber in a wave-like manner.

However, while mechanical signals have been inferred from indirect observations such as sound recordings, direct visualization of these mechanical signals and their propagation dynamics along muscle fibers remains lacking. Here, we aimed to directly observe the propagation of extending wave or ‘stress wave’ along the fiber originating from the initial local contraction at the end-plate region in isolated frog sartorius muscle at high temporal and spatial resolutions.

## 2 Materials and methods

### 2.1 Ethical approval and compliance

The animal experimental protocol conformed to the “Guidelines for the Proper Conduct of Animal Experiments” (2006) of the Science Council of Japan and was reviewed and approved (No. 2024–027) by the Animal Experiment Committee of Jikei University School of Medicine.

### 2.2 Experimental setup

**Fig. 1:**
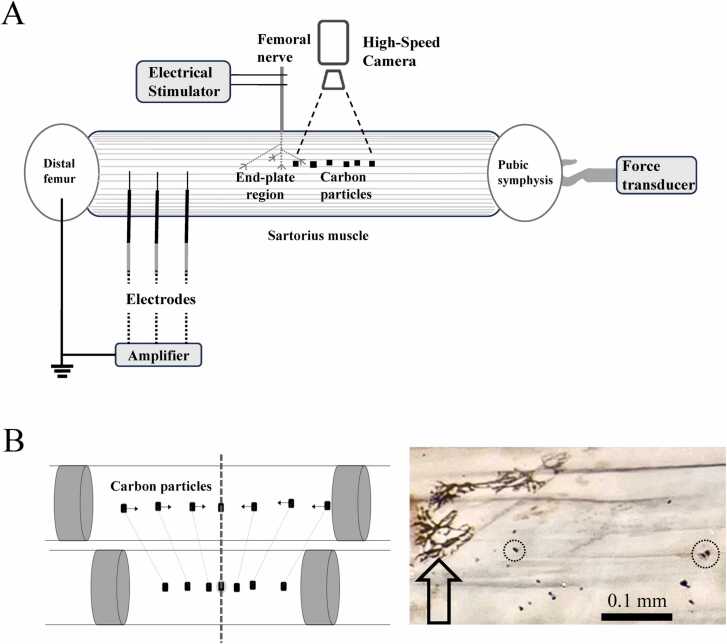
**A.** Schematic diagram of the experimental setup. **B.** Left: Estimation of the location of the end-plate region as the center of symmetry of carbon particle movements. Right: Identification of the end plate region by acetylcholine esterase staining. Arrow: end plates. Circle: carbon particle to be observed on the twitching muscle fiber.

The experimental setup is shown in Fig. 1A. The sartorius muscle (50–55 mm long) of bullfrogs (*Rana catesbeiana*) with bone fragments at both ends and nerves were dissected. The specimen was soaked in Ringer solution (118 mM NaCl, 2.5 mM KCl, 1.8 mM CaCl₂, and 5.0 mM N-[2-hydroxyethyl] piperazine-N′- [2-ethane -sulfonic acid], with the pH adjusted to 7.4 at 20 ℃). The nerve was placed on a pair of stimulation electrodes (Fig. 1A), and square pulses were applied for 0.5 ms from a stimulator (DPS-10 with an isolator DPS-101; DiaMedical, Tokyo, Japan). The pubic bone fragment was attached to a tension transducer (UTA-500GR; Minebea Mitsumi, Tokyo, Japan), which was connected to a strain amplifier (DPM-912B; Kyowa, Tokyo, Japan) for recording. Meanwhile, the femoral bone fragment was fixed by passing two needles through the femoral head. The tension transducer was secured to an actuator (DRSM42RG-04A8AZMK; Oriental Motor, Tokyo, Japan) driven by a controller (AZD-KD; Oriental Motor, Tokyo, Japan). The setup was adjusted to ensure the neuromuscular specimen remained untwisted and was set to the optimal length for generating maximum tension. Microcarbon particles (diameter 10 – 20 µm) were prepared by shaving the graphite core of a 6H pencil (Hi-Uni; Mitsubishi Pencil, Tokyo, Japan) and were randomly attached to the muscle surface. We selected the muscle fibers that twitched at the minimal threshold of nerve stimulation on the muscle surface.

### 2.3 End-plate estimation and electrode setup

The fiber end-plate location was estimated to be the center of symmetry of the movement of the carbon particles on the fiber (Fig. 1B; left). For potential measurements, three platinum wire electrodes (0.3 mm in diameter) were placed along the fiber on the femoral side of the end-plate region at intervals of 2–3 mm. The more detailed positions of the electrodes were confirmed and measured using microscope images with a spatial resolution of 14.5 µm per pixel. A reference electrode was placed on the periosteum of the distal femoral bone fragment. Electrode-recorded potentials were amplified (MEG-2100; Nihon Kohden, Tokyo, Japan) and digitized using a PowerLab26T data acquisition system (AD Instruments, Sydney, Australia) at a 4 kHz sampling rate. Action potential conduction velocity was calculated based on the time delay of the spike potential peaks across the electrode pairs.

### 2.4 High-speed camera setup and resolution

The carbon particles on the pubic side of the twitching fiber were video-recorded under a stereomicroscope at 30,000 frames per second using a high-speed camera (Phantom VEO-E 310 L; Vision Research, Wayne, NJ, USA) and analyzed using ImageJ software (National Institutes of Health, Bethesda, Maryland, USA). The overall spatial resolution of the displaced carbon particles on the video images was 10 µm, which was slightly smaller than the pixel size (14.5 µm) because of the processing of the center of intensity gravity over the pixel array.

### 2.5 End-plate identification and sarcomere measurement

At the end of the experiment, the end-plate location on the fiber was histochemically identified through acetylcholinesterase staining using the Acetylcholinesterase Rapid Staining Kit (MBL, Nagoya, Japan; Fig. 1B; right) [10]. Finally, the specimens were pre-fixed in a half-Karnofsky fixative (2.5 % glitaraldehyde-2 % paraformaldehyde in 0.1 M phosphate buffer) at the optimal length and post-fixed in 2 % glutaraldehyde to measure the sarcomere spacing using Helium-Neon laser diffraction. Specimens were analyzed within 1 h of animal euthanasia.

### 2.6 Statistical analyses

Data were expressed as mean ± standard deviation, and the *t*-test and one-way analysis of variance were used for inter-group comparisons. Tukey’s multiple comparison test was performed when a statistically significant difference was observed using these methods. Statistical significance was set at p < 0.05. Analyses were conducted using the statistical analysis software R version 4.4.1 (https://www.r-project.org/).

## 3 Results

### 3.1 Propagation of action potentials and extension

**Fig. 2:**
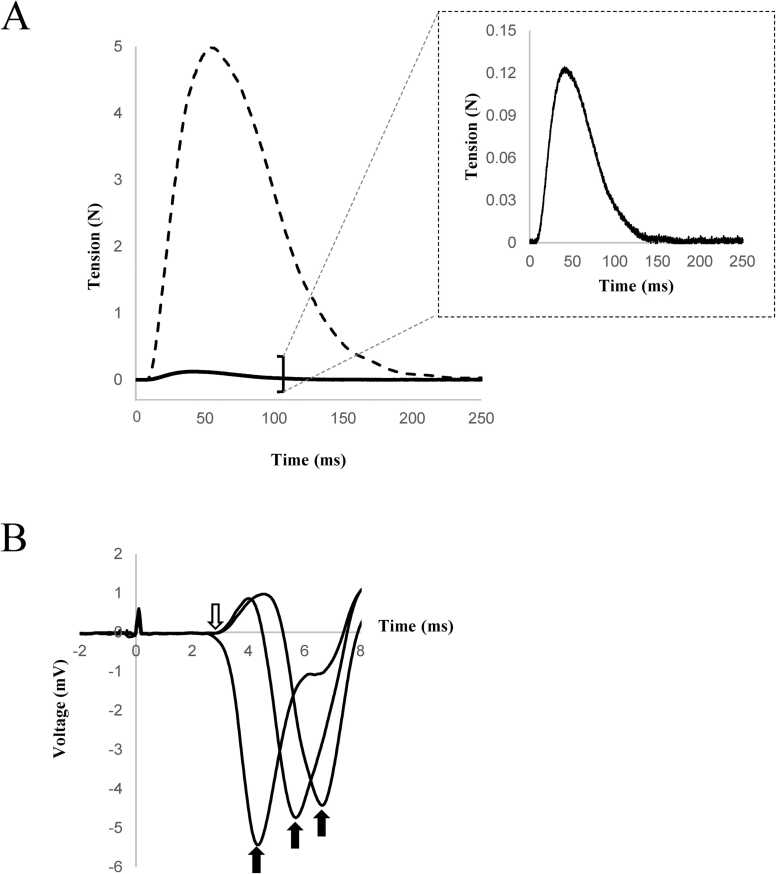
**A.** Representative tension recording with nerve stimulation at the maximal and threshold levels. Solid line: threshold-level tension. Dashed line: maximal tension. An enlarged view of the threshold-level tension is shown in the inset. **B.** Recorded action potentials on a twitching fiber. Open arrow: Presumed onset of action potential at the end-plate region. Closed arrow: peak of potential spike representing the passing of action potential over the electrode. The abscissa indicates time elapsed from nerve stimulation.

To detect the propagation of mechanical waves along the twitching muscle fiber at the optimal length (sarcomere spacing 2.31 ± 0.06 µm), we applied a minimal electrical pulse to the nerve for 0.5 ms to activate a few motor units. The induced twitch force was 1.80 – 3.02 % of the maximal twitch force (Fig. 2A).

The action potential and local contraction were propagated from the end-plate region along the twitching muscle fiber. We detected the propagation of action potentials at three sites on the femoral side of the fiber end-plate region (Fig. 1A). A large spike in electrical potential, representing the propagation of action potentials over the probe electrode, delayed by the distance from the end-plate region, was observed, indicating the propagation of action potential at 2.9 m·s⁻¹ on the fiber (Fig. 2B). The rate was 3.04 ± 0.31 m·s⁻¹ in seven specimens.

We attached carbon particles to the muscle surface to detect the propagation of mechanical waves along the muscle fibers. The particle serves as a readily observable marker for detecting the displacement of the underlying muscle fiber induced by the mechanical stimulus. With the minimal stimulation on the nerve, particle displacement was the largest on the twitching fiber, and it decayed exponentially with a distance from the fiber at a length constant of 0.47 mm, confirming that the displacement represents the movement of the muscle fiber lying directly underneath the particle.

**Fig. 3:**
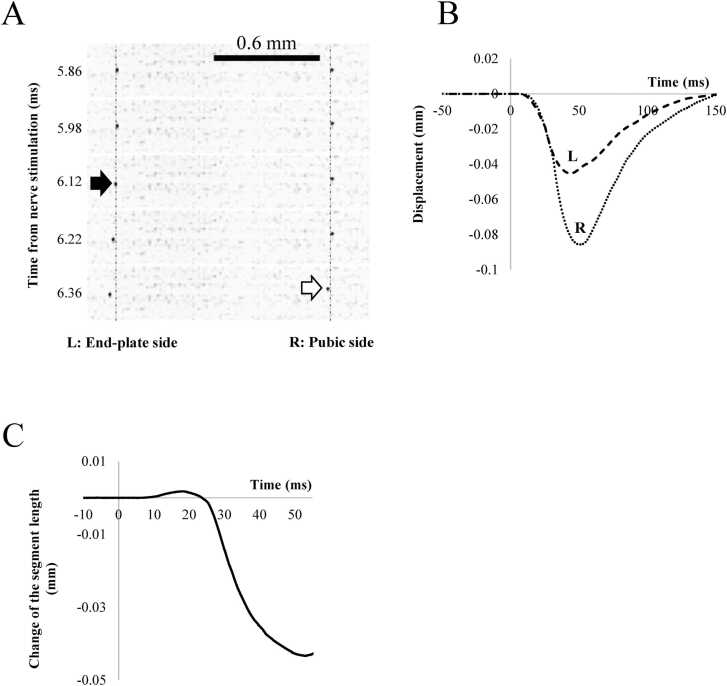
**A.** Images of carbon particles L (located closer to the end-plate region) and R (located farther from the end-plate region). Initial extending displacement were detected at arrows. **B.** The displacement of representative carbon particles L and R on the twitching fiber, quantified as the distance moved along the fiber’s longitudinal axis relative to their initial positions. **C.** Separation between L and R indicating the initial extension and the following shortening of the fiber segment. The abscissa indicates time elapsed from nerve stimulation.

Fig. 3A shows the initial displacement of the particles in the image data, and the particle closer to the end-plate region exhibited an earlier onset of movement, indicating that the fiber segment between the two particles was initially stretched. Fig. 3B shows the quantified displacement along the fiber’s longitudinal axis. These particles initially moved toward the end-plate region after the nerve stimulus and returned to their original positions within approximately 150 ms. Subsequently, the fiber segment between the particles underwent significant shortening, as shown in Fig. 3C.

**Fig. 4:**
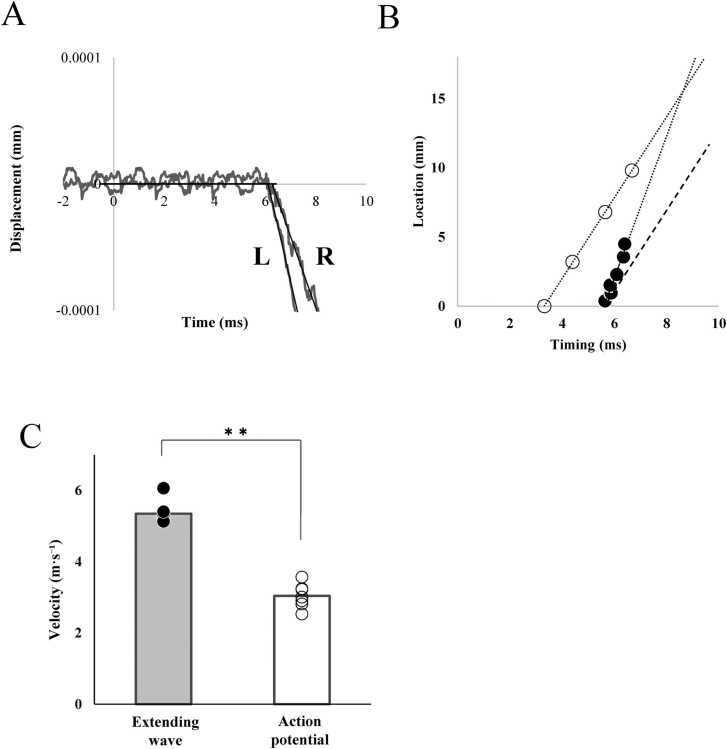
A. Displacement of the carbon particles at L and R along the fiber direction. Onset of the displacement was extrapolated assuming uniform motion at the initial phase. Abscissa indicates time elapsed from nerve stimulation. B. Spatiotemporal propagation diagram of the peak of the potential spike (open circles) and the extrapolated onset of the initial displacement along the fiber (closed circles). Ordinate: the location of the particles and the recording electrodes as the distance from the end-plate region. Abscissa: the extrapolated timing of the displacement onset and the potential peak as the time elapsed from nerve stimulation. The dotted lines indicate the extrapolated trajectories of the extending wave and the action potential propagation. The dashed line indicates the estimated propagation of contraction, assuming a constant delay from the potential peak. C. Propagation velocities of the extending wave (closed) and action potentials (open) in 7 muscle specimens, each from a different animal. **p < 0.01, indicating a significant difference between groups.

The timing of the initial displacement in Fig. 3A was recorded at 6.12 ms after applying the electrical stimulus to the particle closer to the end-plate region (black arrow in Fig. 3A). Further particles exhibited an initial displacement at 6.36 ms after the nerve stimulus (white arrow in Fig. 3A). As the particles were initially 1.2 mm apart, the propagation velocity of the displacement was estimated to be approximately 5 m∙s^−1^. For a more precise analysis, the onset of the initial displacement was extrapolated (Fig. 4A), assuming a uniform linear motion at the initial part of the displacement. The onset of the particles on the twitching fiber shown in Fig. 4A was plotted on a spatiotemporal diagram as closed circles (Fig. 4B). In this study, we focused on the phase where the distance between adjacent particles increases, excluding the subsequent shortening phase and defined the extending wave as the propagation of the initial displacement of the particles. This definition is based on the observation that the initial displacement of the particles reflects the stretching of the fiber segment between them, which is then followed by a shortening phase. The propagation velocity of the extending wave was estimated based on the onset of this stretching phase. Because the observed particles were initially displaced toward the end-plate region and were successively observed at increasing distances from the end-plate region, the linear arrangement of the plots indicates the propagation of an extending wave. As shown in Fig. 4B, the propagation rate of the extending wave was estimated to be 5.13 m·s⁻¹ in this fiber based on the slope of the regression line. The conduction of action potentials, recorded as the peak of the spike potential (Fig. 2B), was also plotted on the same spatiotemporal diagram. The postulated contraction propagation along the fiber is indicated by a dashed line (see Discussion).

The measurement results for the seven specimens are shown in Fig. 4C. The propagation rates of the extending wave ranged from 5.13 to 6.06 m∙s^−1^ with an average of 5.35 ± 0.33 m∙s^−1^. Linear extrapolation of the extending wave propagation and the conduction of action potential indicates that the extending wave was generated 2.72 ± 0.30 ms after the action potential was generated at the end-plate region, and its rapid propagation would overtake the slower propagation of action potential at 23 mm from the end-plate region.

### 3.2 Mediator of the extending wave

To test whether the extending wave is linked to the local activation of the fiber or is a passive mechanical influence transmitted along the fiber, the muscle surface was locally compressed using a stainless-steel rod with a diameter of 0.4 mm. Compression was applied incrementally using a manipulator, increasing by 0.1 mm from the position where the rod made contact with the muscle surface, until the minimal compression required to block the propagation of the contraction wave was reached. Although stainless steel is electrically conductive, the electrode placed near the pubic end of the muscle indicated that compression did not affect the conduction of action potentials.

**Fig. 5:**
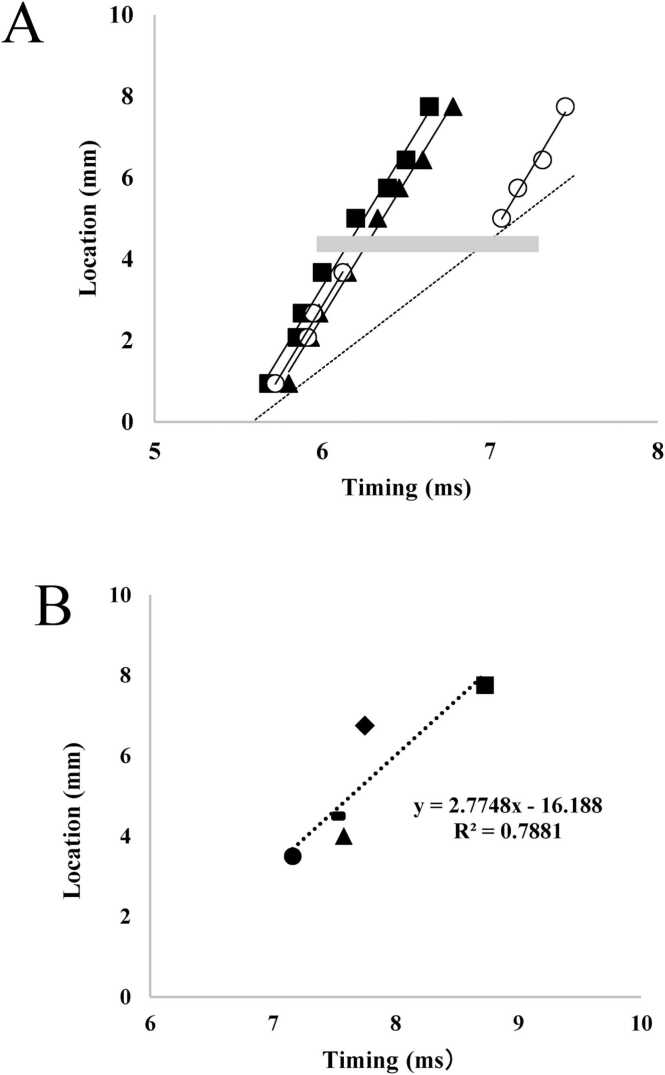
A. Spatiotemporal propagation diagram of the displacement onset of the particles before (closed square), during (open circle), and after (closed triangle) the compression at the location indicated by the gray bar in the figure. The location of each particle is expressed as the distance from the end-plate region, and the timing is expressed as the time elapsed from nerve stimulation. During compression, the dashed line indicates the estimated propagation of contraction wave based on action potential conduction. **B.** Spatiotemporal distribution diagram of the timing of regeneration of the propagating displacement at the location of compression expressed as the distance from the end plate region. Timing is expressed as the time elapsed from nerve stimulation. 5 specimens were obtained, each from a different animal.Specimens are indicated as follows: Specimen 1 (◆), Specimen 2 (▲), Specimen 3 (▬), Specimen 4 (•), Specimen 5 (■).

The propagation of the extending wave was blocked at the compressed site, indicating that propagation was mechanically transmitted along the fiber. In addition, the propagation of the extending wave restarted in the compressed area with a time delay (Fig. 5A).

To evaluate the effect of local compression on the propagation velocity of the extending wave, the velocity was measured before and after compression. Subsequently, the results were compared with the 95 % confidence interval of the propagation velocity observed before the application of local compression. Spatiotemporal analysis indicated that the propagation velocity of the extending wave was 6.84 and 6.92 m·s⁻¹ at the end-plate and pubic sides of the compression, respectively, with both within the 95 % confidence interval (6.15 – 7.36 m·s⁻¹) of the propagation velocity of the extending wave observed before the application of local compression (Fig. 5A). Observations of local compression at various sites showed similar results (Table 1).

**Table 1:** Propagation velocities at the proximal and distal regions during compression, compared with the 95 % confidence interval of the values before compression.

		Before compression	During compression	
Specimen	Location* (mm)	95 % Confidence Interval (m·s⁻¹)	End-plate side (m·s⁻¹)	Pubic side (m·s⁻¹)
**1**	6.75	5.78 - 6.79	6.10	6.31
**2**	4.02	5.40 - 6.37	5.80	6.25
**3**	4.52	6.15 - 7.36	6.84	6.92
**4**	3.55	4.79 - 7.26	6.63	6.73
**5**	7.75	4.72 - 5.73	4.91	5.56

* Location of the compression site expressed as the distance from the end-plate region.

Fig. 5B shows the restarting timing of the extending wave at various compression sites in a spatiotemporal diagram. A linear relationship indicates that the restarting source of the extending wave propagated at a velocity of 2.77 m·s⁻¹ (0.13 – 5.41 m·s⁻¹; 95 % confidence limit) along the muscle fiber. This velocity was close to the propagating velocity of the action potentials described above, indicating that the restarting source of the extending wave was linked to the local activation of the fiber.

### 3.3 Effects of stretch

**Fig. 6:**
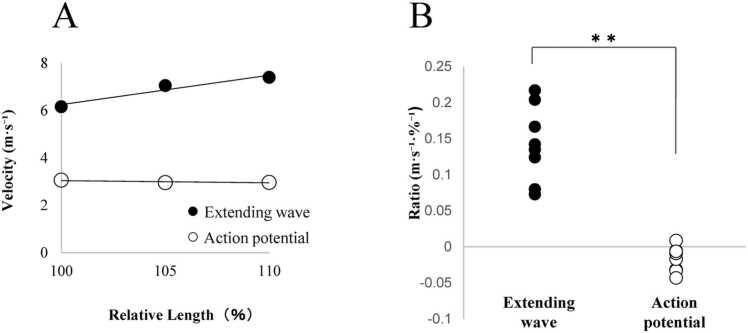
A. The propagation velocity of the displacement onset and peak potential in stretched muscle in a representative specimen. B. The average ratio of the velocity changes to extension extent in 8 specimens, each from a different animal. **p < 0.01, indicating a significant difference between groups.

To study the effects of stretching on the propagation of the extending wave, the muscle specimens were stretched by 5 % and 10 % from their optimal length. The propagation velocity of the extending waves increased in response to muscle stretching, whereas that of the action potentials decreased slightly (Fig. 6). The velocity changes in the extending wave and action potential relative to the stretch extent differed significantly (p < 0.01).

## 4 Discussion

### 4.1 Identification of the end-plate region

The end-plate region was identified as the center of symmetry of the movement of the carbon particles, as confirmed using acetylcholinesterase staining (Fig. 1B). However, because multiple innervations of a single fiber have been reported in frog muscle [11], acetylcholinesterase staining may only confirm the existence of end plates in the supposed area. Therefore, we confirmed our results based on the assumption that the initial synchronous electrical deviation recorded at multiple locations represents the generation of action potentials in the end-plate region (white arrow in Fig. 2B). Based on this assumption, we estimated the position of the end-plate region from the conduction time to the local peak potential and the conduction velocity of the action potential. Supported by this supplemental estimation, we considered that we could successfully identify the location of the end-plate region histochemically with an error of < 0.1 mm.

### 4.2 Propagation of action potentials

The observed propagation velocity of the action potential (3.04 ± 0.31 m·s⁻¹ at 24 ℃) is consistent with a reported value of frog sartorius muscle (0.85 m·s⁻¹ at 7 ℃) [12] considering a temperature coefficient of 2 [13].

The extrapolated timing of action potential generation in the end-plate region was 3.2 ms after the nerve stimulation. Because the distance from the stimulation site on the sciatic nerve to the end-plate region on the observed twitching muscle fiber was approximately 55 mm, the averaged nerve conduction velocity was 20 m·s⁻¹, assuming a synaptic delay of 0.3 ms at 24 ℃ [14], [15]. Because the conduction velocity of the frog sciatic nerve is reportedly 25 – 42 m·s⁻¹ [16], a significant fraction of the conduction distance of 55 mm would be thin axonal branches with low conduction velocity. The thin branches possibly weave through the muscle, and the distance from the entry point of the nerve trunk to the sartorius muscle in the end-plate region of the observed fiber was 20 – 30 mm in the present study.

The varied activation timing owing to slow conduction through thin branches of various lengths should be considered when analyzing these results. However, because the maximal displacement of the observed particles decayed exponentially from a twitching fiber with a length constant of 0.47 mm (data not shown), we hypothesize that the fibers activated with minimal stimulation intensity were distributed sparsely enough to affect only nearby carbon particles.

### 4.3 Generation and propagation of extending waves

Analysis of the displacement of carbon particles on a twitching muscle fiber indicated the rapid propagation of the mechanically extending wave along the muscle fiber. The contraction initially induced in the end-plate region was the most probable source of the extending wave. The propagation velocity of the extending wave was higher than that of the action potential. The lag between the extrapolated timings of the generation of the action potential and the extending wave (2.72 ms; Fig. 4B) represents the time required for local excitation–contraction coupling, rather than an artifact due to differences in measurement positions. This is because the observed delay aligns well with previously reported excitation–contraction coupling delays [17], and it also closely matches the delay of detectable contractile force development following direct muscle fiber stimulation (2.47 ms in the present study; data not shown).

The generation of the extending wave follows a sequential process: first, the activation of voltage-dependent channels induces the generation of an action potential at the end-plate. Then, the action potential propagates into the T-tubule system near the end-plate region, leading to the release of Ca²⁺ from the sarcoplasmic reticulum, which triggers the contraction of nearby sarcomeres. The contraction of these sarcomeres pulls the adjacent Z-lines closer. This local contraction generates mechanical tension stretching longitudinal elastic elements, including connectin/titin and the cytoskeletal system, to cause mechanical strain in the surrounding myofibrils. The strain propagates as a wave-like deformation along the muscle fiber, leading to the formation of the mechanically extending wave. Subsequently, the contraction wave propagates along the muscle fiber following the extending wave.

The faster propagation of the extending wave (5.35 ± 0.33 m·s⁻¹) followed the slower action potential along the muscle fiber from the end-plate region with decreasing delay and eventually overtook it near the distal end of the specimen (Fig. 4B). This indicates that the extending wave propagated through an excited but non-contracted region of the fiber. Because the extending wave propagates at a constant velocity along the fiber, the excited but non-contracted part of the muscle fiber appears to have uniform mechanical properties for the propagation of the extending wave. To ensure the robustness of this interpretation, we further evaluated whether the observed difference in propagation velocities could be attributed to measurement limitations. Regarding the action potential, the estimated conduction velocity error based on spatial and temporal resolution was +0.26 m·s⁻¹. Furthermore, when assuming positional uncertainty corresponding to the width of the electrode contact, the maximum estimated error increased to +0.56 m·s⁻¹. For the extending wave, the estimated error in propagation velocity was −0.25 m·s⁻¹. The directions of these errors were intentionally chosen to represent a conservative estimation scenario, where an overestimation of the action potential conduction velocity (+) and an underestimation of the extending wave propagation velocity (−) would minimize the observed difference. Even after accounting for these maximum possible errors, the propagation velocity of the extending wave remained higher than the conduction velocity of the action potential, supporting the validity of this comparative result. Furthermore, as shown in Fig. 6, the slight decrease in the conduction velocity of action potentials contrasting to the increase in the propagation velocity with muscle stretch further supports the notion that the propagation of extending waves is essentially independent from that of action potentials.

In this study, extending waves were primarily observed on the surface of muscle fibers, while the propagation characteristics within the fibers remain largely unclarified. Theoretically, since the conduction velocity of action potentials in the T-tubule is slower than that along the sarcolemma (muscle fiber membrane), it is expected that the generation of extending waves inside the fiber is delayed depending on the distance from the surface. Furthermore, the propagation of extending waves within muscle fibers may be differentially influenced by the mechanical properties of surrounding fibers and the connective tissues that structurally link them, including the endomysium. Considering these issues, the interrelationship of the propagational property of the action potential and the extending wave could be altered deep inside the fibers. This alteration may affect the observed property of the extension wave on the surface partly via parallel elastic components.

### 4.4 Extending wave and fiber elasticity

The extending wave generated by the early contraction at the end-plate region is a longitudinal stress wave transmitted along the fiber through elasticity. Because the elastic contribution of sarcolemma is reportedly negligible until the fibers are extended to a sarcomere spacing of 2.8 µm [18], the extending waves observed at the optimal sarcomere spacing of 2.31 ± 0.06 µm, and the 5 % and 10 % stretched states was primarily mediated by myoplasmic elastic components such as connectin/titin in sarcomere. Stretches of 5 % and 10 % have been reported to cause a gradual increase in the elasticity of the myoplasm, as evidenced by a hyperbolic increase in the resting tension with sarcomere spacing [19]. The observed increase in the extending wave velocity suggests 80 % and 200 % increases in the myoplasmic resting tension with 5 % and 10 % stretches, respectively. These values are close to the observed increases (71 % and 197 %, respectively) in the resting tension of our muscle specimens (data not shown). Thus, the increase in myoplasmic resting tension with fiber extension likely enhances the efficiency of force transmission, as the increased elastic components contribute more effectively to the propagation of mechanical signals along the muscle fiber.

### 4.5 Mechanical signals of a twitching motor unit

As indicated by the displacement of the carbon particles shown in Fig. 3C, the initial fiber extension was followed by successive shortening of the fibers. From the well-documented isovolumic behavior of skeletal muscle sarcomeres [20], the preceding extending wave would accompany thinning, and the subsequent shortening would accompany the thickening of the muscle fibers. Switching from extension to shortening would accompany switching from thinning to thickening of the fiber. Successive thinning and thickening would cause successive decreases and increases in pressure, causing vibrations around the muscle surface. In humans, the vibration generates mechanical signals or sounds on the skin surface covering the twitching muscle fibers, as proposed by Morimoto and Takemori [9].

The restarting source of the extending wave at the compressed sites (Fig. 5) propagated at a velocity almost identical to that of the action potential. Therefore, we considered the source to represent the propagating contraction wave following the propagation of the action potential, with an almost constant delay necessary for excitation-contraction coupling (2.72 ms; Fig. 4B). As the extending wave propagates faster than the contraction wave (Fig. 4B), the time interval between the start of thinning and the switch to thickening increases with the distance from the end-plate region, as reported by Morimoto and Takemori [9].

### 4.6 Implication to sports medicine and rehabilitation

The propagation of the extending wave and the switching from extension to shortening may cause instantaneous mechanical strain that damages the contracting and neighboring muscle fibers. In addition, the propagation of the extending and contracting waves reflects the elastic responses of muscle fibers under physiological conditions. Practical assessment of these waves can be achieved by recording mechanical signals (muscle sounds) from the target muscle on the skin surface, as proposed by Morimoto and Takemori [9]. Similarly, controlled twitching of the target muscles for assessment can be induced percutaneously. Therefore, evaluating the extending and contracting waves targeted in this study may be broadly applicable to various practices in the fields of sports medicine and rehabilitation, including preventing muscle injury, monitoring recovery, and optimizing motor performance.

For example, assessment of these waves may also be applicable as a means of visualizing and understanding the condition of fatigued or damaged muscles. Previous studies using ultrasound and shear wave elastography, which are widely used in clinical settings, have demonstrated increased muscle elasticity under such conditions [21], [22], [23], and this change has been reported to serve as an indicator of muscle recovery or damage. However, the evaluation of muscle elasticity using ultrasound and shear wave elastography, may have limited utility in determining the actual functional performance of muscles. This is because physiological force transmission in muscle fibers occurs primarily in a unidirectional manner along the fiber, and the viscosity of the fibers interferes with their elastic properties.

As another example of clinical application, assessing the extending wave could contribute to preparing the muscle’s elastic components, such as connectin/titin, for subsequent smooth and efficient contraction. This preparatory effect may resemble the brief pre-movement muscle elongation observed in functional activities, including jumping, kicking, or walking. If the characteristics of extending waves can be linked to specific mechanical responses in muscle tissue, it may become possible to prescribe appropriate movement strategies aimed at minimizing strain-induced damage.

According to Fig. 3C, the change in sarcomere length during the propagation of the extending wave was 0.13 %, suggesting that a single twitch does not stretch connectin/titin to the extent that the anchor between connectin/titin and the thick filament becomes dislodged [24]. However, dislodgement of connetin/titin may still occur under certain conditions, such as an extended initial sarcomere length or increased local heterogeneity in elastic properties. Furthermore, the elasticity of connectin/titin is known to be modified during or after vigorous muscle contraction [25], [26]. These changes could alter the propagation characteristics of the extending wave to influence overall muscle performance.

Furthermore, examining the relationship between particle displacement and tension development may provide insights into how these mechanical waves contribute to force generation and motor output. Notably, the onset of tension approximately coincided with the arrival of the extending wave, suggesting its potential involvement in the initiation of whole-muscle force development (data not shown). This observation indicates that further investigation of wave propagation dynamics may help estimate the factors contributing to the time delay between neural excitation and tension development.

### 4.7 Limitations of the study and future perspectives

In the present study, we used the sartorius muscle of bullfrogs because of its size and the relatively long vital period post-dissection. To apply the experimental results to analyze the mechanical signals of the human skeletal muscle, observation of animals that are more closely related to humans under more physiological conditions is awaited. Also, to better understand how the extending wave contributes to physiological phenomena in vivo, it will be important to investigate how these responses differ under tetanic contractions, as the present study was limited to single twitches.

Moreover, we inferred the changes in fiber thickness accompanying the propagation of the extending wave based on the assumption of the isovolumic behavior of sarcomeres. Direct observation of fiber thickness during transient extension and shortening would provide further validation of the mechanisms underlying the mechanical signals recorded from twitching muscle.

Extending waves were primarily observed on the surface of muscle fibers in this study, while the mechanical behavior within the fibers remains unmeasured. To elucidate the propagation characteristics of extending waves inside muscle fibers, further investigations employing alternative imaging techniques, combined with mathematical modeling, are required.

## CRediT authorship contribution statement

**Nakahara Naoya:** Writing – review & editing, Visualization, Validation, Software, Resources, Methodology, Formal analysis, Conceptualization. **Morimoto Shigeru:** Writing – review & editing, Supervision, Methodology, Formal analysis, Conceptualization. **Yamaguchi Maki:** Writing – review & editing, Writing – original draft, Supervision. **Hirano Kazuhiro:** Writing – review & editing, Conceptualization. **Takemori Shigeru:** Writing – review & editing, Supervision, Project administration, Conceptualization. **Hayashi Tomonori:** Writing – review & editing, Writing – original draft, Visualization, Investigation, Funding acquisition, Formal analysis, Data curation.

## Funding

This study was supported by a graduate school research grant from 10.13039/501100007962Jikei University and JSPS KAKENHI (Grant Numbers 22K09434). The funding sources had no role in the study design, data collection, analysis, interpretation, or manuscript preparation.

## Declaration of Competing Interest

The authors declare that they have no known competing financial interests or personal relationships that could have appeared to influence the work reported in this paper.

## Data Availability

The datasets generated and analyzed during this study, including high-speed video recordings and displacement data of carbon particles, are available from the corresponding author on reasonable request.
